# The interplay of psychological resilience and adolescent mobile phone addiction in Henan province, China: insights from latent class analysis

**DOI:** 10.3389/fpubh.2024.1386500

**Published:** 2024-06-20

**Authors:** Jun Xiao Wu, Lin Jia, Yan Li, Qian Liu, Ying Ying Zhang, Jin Zhang, Yan Rong Jia, Zhen Fan

**Affiliations:** ^1^School of Nursing, Nanyang Medical College, Nanyang, Henan, China; ^2^Faculty of Business, Economics and Law, The University of Queensland, St Lucia, QLD, Australia; ^3^Emilio Aguinaldo College, Manila, Philippines

**Keywords:** adolescent, resilience, mobile phone addiction, DASS-21, ROC curve, cut-off point, latent class analysis

## Abstract

**Background:**

The aim of this study was to classify distinct subgroups of adolescents based on the severity levels of their mobile phone addiction and to investigate how these groups differed in terms of their psychosocial characteristics. We surveyed a total of 2,230 adolescents using three different questionnaires to assess the severity of their mobile phone addiction, stress, anxiety, depression, psychological resilience, and personality. Latent class analysis was employed to identify the subgroups, and we utilized Receiver Operating Characteristic (ROC) curves and multinomial logistic regression for statistical analysis. All data analyses were conducted using SPSS 26.0 and Mplus 8.5.

**Methods:**

We classified the subjects into subgroups based on their mobile phone addiction severity, and the results revealed a clear pattern with a three-class model based on the likelihood level of mobile phone addiction (*p* < 0.05). We examined common trends in psychosocial traits such as age, grade at school, parental education level, anxiety levels, and resilience. ROC analysis of sensitivity versus 1-specificity for various mobile phone addiction index (MPAI) scores yielded an area under the curve (AUC) of 0.893 (95% CI, 0.879 to 0.905, *p* < 0.001). We also determined diagnostic value indices for potential cutoff points ranging from 8 to 40. The optimal cutoff value for MPAI was found to be >14, which corresponded to the maximum Youden index (Youden index = 0.751).

**Results:**

The latent classification process in this research confirmed the existence of three distinct mobile phone user groups. We also examined the psychosocial characteristics that varied in relation to the severity levels of addiction.

**Conclusion:**

This study provides valuable insights into the categorization of adolescents based on the severity of mobile phone addiction and sheds light on the psychosocial characteristics associated with different addiction levels. These findings are expected to enhance our understanding of mobile phone addiction traits and stimulate further research in this area.

## 1 Introduction

In the past decade, the number of adolescents that use the internet has grown enormously. Latest figures show that approximately 5.3 billion of the Earth’s 8 billion people used the internet in 2022, or roughly 66% of the world’s population. At the same time, three quarters of the population aged 10 years and over owned a mobile phone. The statistics further show that young people are the driving force of connectivity, with 75% of 15–24 year olds now regularly online (1). According to the 51st Statistical Report on Internet Development in China published in December 2022, the proportion of individuals using the internet via mobile phones reached 99.8%. Among them, users aged 10–19 now account for 14.3% of the total number of users (2). In mainland China, around 80% of teenagers possess mobile phones, with nearly 40% of them using mobile phones without restrictions (3). While advances in the availability of the internet have brought many benefits to its users, its widespread adoption has also led to various types of harm, particularly to adolescents who are in a rapid stage of physical and psychological development and are susceptible to external influences. Therefore, excessive mobile phone usage may lead to unhealthy physical and psychological outcomes in teenagers.

Many studies have identified the predictors of mobile phone addiction: excessive use of mobile phones can contribute to negative physiological and psychological consequences, such as headaches and sleep disorders (4), neuroticism (5), trouble concentrating (6), and stress, anxiety, and depression (7). Although some studies have established the relationship between mental health and mobile phone addiction (8–10), all have ignored the within-class correlation between mobile phone addiction and other phenotypes. Therefore, the present study used latent class analysis to narrow the research gap in the field of psychological resilience and mobile phone addiction.

## 2 Literature review

### 2.1 Mobile phone addiction

Mobile phone addiction can be classified as a behavioral addiction, exhibiting four typical features: compulsion, functional impairment, tolerance, and withdrawal (11). The addictive nature of mobile phones aligns with behavioral addiction, a disorder characterized by behavioral symptoms linked to pleasure and craving (12). Mobile phone addiction has been referred to as one of the most important social problems of the modern era (13). Problematic mobile phone use has been related to cognitive impairments (14) and depression (15). Addiction to mobile phones can be described as a secretive disease that as it results in damaging psychological health and impacting the quality of life, it is only natural to try to understand the behaviors associated with it (16).

### 2.2 Psychological distress

Psychological distress includes symptoms of stress, anxiety, and depression. Significant psychological distress indicates impaired mental health and may reflect common mental disorders such as depression and anxiety disorders (17).

Severe psychological distress is connected with problematic health behaviors such as extreme alcohol consumption, smoking, and a propensity to commit (or an attempt to commit) suicide (18). Psychological distress among students is influenced by sociodemographic and contextual factors, academic-related factors such as performance and achievement at school or college, as well as personality and individual differences (18). Many studies have reported a high prevalence of psychological distress among adolescents. Prevalence rates of 40% have been recorded in China (19), 54% in Saudi Arabia (20), and 20.6% in Tanzania (21). Academic stress and adverse life events may account for much of this high prevalence of psychological distress among adolescents (22). However, a growing number of adolescents now spend a good deal of their time on their mobile phones, leading to an increasing incidence of mobile phone addiction. Mobile phone addiction has been regarded as an important cause of a variety of psychological and behavioral problems (23). Adolescents immersed in the content of their mobile phones may experience added psychological distress due to poor sleep quality and a tendency to procrastinate resulting from excessive or uncontrolled phone usage. The extent of disruption caused by mobile phone usage on anxiety and sleep quality exacerbates the deterioration of mental health, increasing levels of psychological tension and physiological agitation. This can have negative repercussions, leading to stress and poor health outcomes.

### 2.3 Psychological defensive features

“Defense mechanisms” are a psychological process, typically accompanied by a behavioral response, used to cope with challenging situations, to manage conflicts, and to safeguard the basic functioning from disturbances caused by distressing, painful, and unacceptable thoughts, feelings, and experiences (24). Psychological protection plays a crucial role in the process of forming the individual and the individual’s needs. These mechanisms shield individuals from negative emotional experiences, facilitate the maintenance of psychological equilibrium, resolve interpersonal conflicts, and occur at the unconscious and subconscious levels of the psyche. Adolescent crisis is one of the most intricate issues within individual development. Adolescents are in a continuous state of adapting to physical and physiological changes, undergoing what is often termed a “hormonal storm.” In its most general form, it can be said that adolescents appear to be consistently under stress, and attempts at restoring personal equilibrium should be considered a central feature of adolescence.

The psychological resilience of adolescents, as one of the psychological defenses, has been confirmed by numerous researchers (25–27).

Cong et al. (28) verified that heightened psychological resilience may reduce the rate of suicide ideation in adolescents. Resilience has been related to decreased psychological distress and to increased positive experiences (29). Resilience can enhance self-assessment and recognized social support and thus favor the high level of emotional regulation skills in adolescents (30). Moreover, psychological resilience serves as a protective factor against smartphone addiction. Zhao et al. (7) revealed that psychological resilience has a significant negative impact on mobile phone addiction, that is, the stronger the psychological resilience, the less likely the mobile phone addiction. Resilience appears to negatively predict mobile phone addiction among minority preparatory students (31).

## 3 The present study

Based on our literature review, the present study focused on the relationship between mobile phone addiction and the unique characteristics of addicted mobile phone users. This study performed Latent Class Analysis (LCA) within the adolescent sample to identify subgroups representing mobile phone addiction and high-risk usage based on the severity of an individual’s mobile phone usage. Then, cross-sectional comparisons were conducted on the psychological characteristics of each group, leading to an estimate of the ratios of these groups in the population.

## 4 Materials and methods

### 4.1 Participants

In this study, 2,300 adolescents from middle schools and high schools in Henan province of China were asked to complete the questionnaires from June 1 to August 30, 2022. In total, 2,230 valid questionnaires were procured, with an effective rate of 96.96%. Among the respondents, the mean age was 14.92 years (SD = 1.867, range 12–19 years), including 962 boys (43.1%) and 1,268 girls (56.9%). Voluntary participation was highlighted in an announcement. The questions of the survey covered the background and confidentiality information of this study, and participants’ privacy was protected. The participants completed the questionnaires at their own school and no incentives were provided. Incomplete questionnaires were excluded from this study.

### 4.2 Measurement of structures

#### 4.2.1 DASS-21

The DASS-21 was used to evaluate t*he* key symptoms of depression, anxiety, and stress (32) and collected information referred to the week prior to completing the questionnaire. The DASS-21 has been proven to have competent psychometric properties and is equivalent to other accurate questionnaires. This scale is classified into a 4-Likert reply from 0 to 3, where 0 = Nothing and 3 = Most of the time. This self-report instrument was designed to assess DASS, and includes three subscales: (1) the stress subscale, which measures tension, agitation, difficulty relaxing, and negative effect; (2) the anxiety subscale, which assesses autonomic arousal, skeletal musculature effects, situational anxiety, and subjective experience of anxiety arousal; and (3) the depression subscale, which measures hopelessness, dysphoria, lack of interest, self-deprecation, and inertia. The reliability coefficients of depression, anxiety and stress were 0.82, 0.82 and 0.79, respectively. The Cronbach’s alpha of the total scale was 0.89.

#### 4.2.2 MPAI

The MPAI has been used to identify the symptoms of mobile phone addiction among adolescents in Hong Kong (33). The scale includes 17 items and 4 subscales answered on a five-point Likert scale (5 = Always; 4 = Often; 3 = Occasionally; 2 = Rarely; and 1 = Not at all). The 4 subscales included “Inability to control craving”; “Anxiety and feeling lost”; “Productivity loss”; “Withdrawal and escape.” The Chinese version of the MPAI has had good reliability and validity when used with students (34, 35). The higher the score indicates a higher degree of mobile phone addiction. The Cronbach’s alpha of scale was 0.886. In addition, items 3, 4, 5, 6, 8, 9, 14, and 15 were designed to be mobile phone addiction screening questions. If participants answered five or more questions with a three or above score, they were regarded as mobile phone addicts. The others were categorized as non-phone addicts.

#### 4.2.3 RSCA

The RSCA was developed by Chinese scholars Hu and Gan (36) according to the process model of the resilience concept and applied to Chinese adolescents. There are 27 items divided into five factors: goal focus, emotion control, positive cognition, family support and interpersonal assistance. This scale is a five-point Likert-type scale from 1 = Completely disagree to 5 = Completely agree. This scale is especially appropriate in the evaluation of Chinese adolescents’ resilience, and is widely used in China under various situations. The reliability of the total scale was 0.85.

### 4.3 Data analysis

All data analyses were performed using SPSS 26.0 and Mplus 8.5 (37). First, descriptive data were received using SPSS 26.0, and correlation variables were calculated using Pearson’s correlations. Second, the present study used Latent Class Analysis (LCA) to identify latent mobile phone addiction class among Chinese adolescents, and the LCA performed by Mplus 8.5 to discover subgroups that displayed similar patterns of individual characteristics (i.e., class homogeneity) and diverse patterns across subgroups (i.e., class separation). LCA is a form of finite mixture modeling which involves clustering, density estimation, and random-effects modeling. The data analysis used fit indices to evaluate the best fitting model, including maximum log likelihood (LL), Akaike information criterion (AIC), the Bayesian information criteria (BIC), the sample-size adjusted Bayesian information criteria (SABIC), entropy (an indication of classification uncertainty), the Lo Mendell and Rubin likelihood ratio test (LMR), and the bootstrap likelihood ratio test (BLRT). Higher entropy values suggest a model that better divides the data into profiles, with values of >0.80 encouraging the minimal uncertainty of the profile classification of adolescents, and a significant BLRT, which compares the fit of the k-profile model with the k-1 profile model. The means of each class were stabilized using the logit values procured from the initial mix model, thus the latent class categorization was not influenced by subsequently added covariates. Finally, the ultimate class model was subsequently returned on the covariates.

## 5 Results

### 5.1 Descriptive statistics

We included 2,230 participants, including 962 boys and 1,268 girls in the final analysis. The proportion of girls was slightly higher than that of boys (56.9% vs. 43.1%, respectively). The mean age was 14.92 years (SD = 1.867, range 12–19 years). There were 1,223 (54.84%) participants from middle school, 1,007 (45.16%) from high school, 161 (7.2%) from one-child families, and 2,069 (92.8%) from multi-child families. The other results are shown in Table 1.

**Table 1:** Demographic profiles and descriptive statistics of the participants.

Dependent variable	Frequency	Percentage
Gender
Boy	962	43.1
Girl	1,268	56.9
One-child
yes	161	7.2
no	2069	92.8
Birth order
1st	1,025	46.0
2nd	1,029	46.1
3rd	176	7.9
Nationality
Han	2,222	99.6
Hui	7	0.4
Miao	1	0.0
Grade
Middle school 7th	163	7.3
Middle school 8th	625	28.0
Middle school 9th	435	19.5
High school 1st	34	1.5
High school 2nd	464	20.8
High school 3rd	509	22.8
Education level of father
Elementary school and below	156	7.0
Middle school	1,275	57.2
High school	572	25.7
Bachelor’s degree	214	9.6
Master’s degree and above	13	0.6
Education level of mother
Elementary school and below	276	12.4
Middle school	1,217	54.6
High school	518	23.2
Bachelor’s degree	206	9.2
Master’s degree and above	13	0.6
Average monthly household income *per capita* (RMB)
≧8,000	156	7.0
6,000 ~ 7,999	226	10.1
4,000 ~ 5,999	455	20.4
2000 ~ 3,999	788	35.3
1,000 ~ 1999	386	17.3
<600 ~ 999	118	5.3
200 ~ 599	67	3.0
<200	34	1.5
Total	2,230	100.0

### 5.2 Correlation analysis of major study variables

The variables correlated with the constructs in Table 2 were less than 0.85. The discriminant validity value (<0.85) was met in the construct correlation (38). We found that some demographic characteristics were related to the participants’ mobile phone addiction. The results of the analysis showed that academic performance, the primary caregiver, parenting style, family style, and financial status were positively correlated with the adolescents’ mobile phone addiction. Conversely, the education level of the parents, the educational level of the primary caregiver, and family history of psychosis were negatively correlated with the adolescents’ mobile phone addiction.

**Table 2:** Correlation analysis of Sample characteristics and MPAI.

	1	2	3	4	5	6	7	8	9
MPAI	1								
Academic performance	−0.162^**^	1							
Education level of father	−0.026	−0.084^**^	1						
Education level of mother	−0.054^**^	−0.113^**^	0.612^**^	1					
Primary caregiver	0.021	0.036	0.018	0.008	1				
Educational level of the primary caregiver	−0.086^**^	−0.087^**^	0.519^**^	0.723^**^	0.005	1			
Parenting style	−0.190^**^	0.075^**^	−0.075^**^	−0.042^*^	0.086^**^	−0.047^*^	1		
Family financial status	0.028	−0.014	−0.127^**^	−0.131^**^	−0.026	−0.118^**^	0.003	1	
Family history of psychosis	−0.069^**^	0.019	−0.024	−0.008	−0.070^**^	−0.056^**^	−0.027	0.019	1

^**^p < 0.01 (two side), ^*^P < 0.05 (two side), sig.

The major study variables correlated with the constructs in the present study. Stress, anxiety, depression, and psychological resilience were correlated with the adolescents’ mobile phone addiction (Table 3). Furthermore, the correlations suggest that there is an association between the psychological variables and mobile phone addiction.

**Table 3:** Correlation analysis of study variables.

	1	2	3	4	5	6	7	8	9
MPAI	1								
Stress	0.415^**^	1							
Anxiety	0.361^**^	0.871^**^	1						
Depression	0.374^**^	0.849^**^	0.858^**^	1					
Goal focus	−0.236^**^	−0.174^**^	−0.156^**^	−0.241^**^	1				
Interpersonal support	−0.275^**^	−0.455^**^	−0.421^**^	−0.464^**^	0.317^**^	1			
Emotional control	−0.475^**^	−0.609^**^	−0.571^**^	−0.567^**^	0.298^**^	0.575^**^	1		
Positive cognitive	−0.081^**^	−0.139^**^	−0.154^**^	−0.196^**^	0.650^**^	0.215^**^	0.187^**^	1	
Family support	−0.285^**^	−0.366^**^	−0.347^**^	−0.395^**^	0.448^**^	0.466^**^	0.420^**^	0.377^**^	1

^**^P < 0.01 (two side), sig.

### 5.3 Detecting latent class

**Figure 1:**
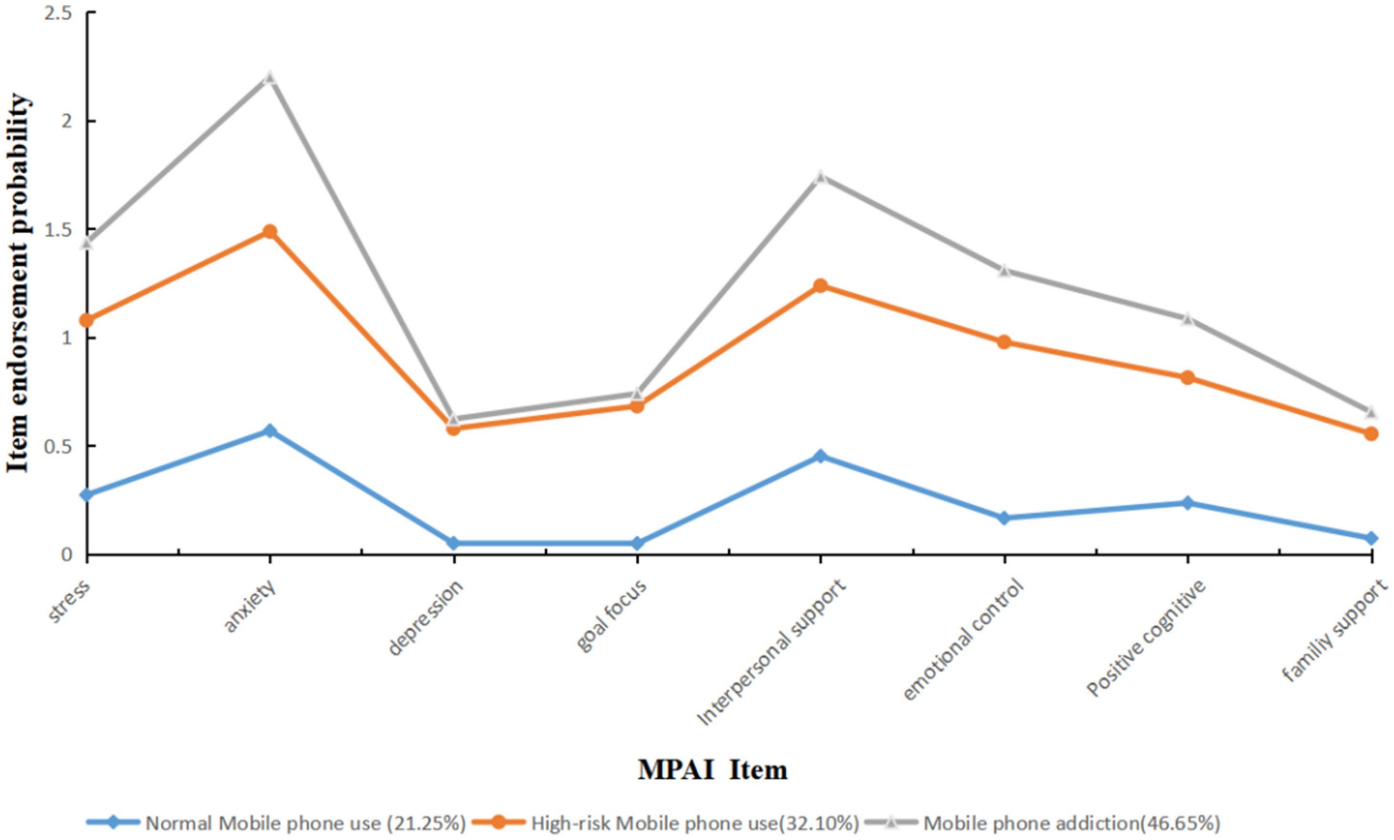
Profiles for 3-class LCA model of MPAI.

This study involves five different model fit statistics that were used to assess which LCA model fits the data best. We detected that AIC values decreased gradually from the 2- to 5-class models, BIC values decreased gradually from the 2- to 4-class models, while BIC did not decrease from the 4- to 5-class model, which the 3-class model had SABIC as well as significant LMR and BLRT. The 3-class model also had the highest entropy score, reflecting the best classification accuracy among other models, suggesting that participants were ranged into three mutually exclusive classes. To sum up, the 3-class model was determined the optimal latent class, therefore we selected the 3-class model as the best fitting model (Table 4). Figure 1 shows the different classes and their means for each mobile phone use subscale. The first profile is called the “normal mobile phone use class” (*n* = 482, 21.50%), and included the least number of adolescents in all subscales of mobile phone use. The opposite class, namely the class in which mobile phone adoption was highest for all subscales of mobile phone use, is called the “mobile phone addiction class” (46.65%). The third class included the most adolescents (*n* = 1,058) and is called the “high-risk mobile phone use class.” Adolescents in this class are characterized by a moderate population in all mobile phone use subscales.

**Table 4:** Fit indices for latent class analysis.

Model	*K*	Log likelihood	AIC	BIC	SABIC	Entropy	LMR *p*-value	BLRT *p*-value
2	65	−11837.617	23805.235	24132.413	23925.954	0.843	0.000	0.000
3	98	−11537.976	23271.952	23765.236	23453.959	0.895	0.000	0.000
4	131	−11407.301	23076.602	23735.992	23319.897	0.804	0.7822	0.000
5	164	−11310.716	22949.431	23774.926	23254.014	0.790	0.7635	0.000

### 5.4 ROC analysis

**Figure 2:**
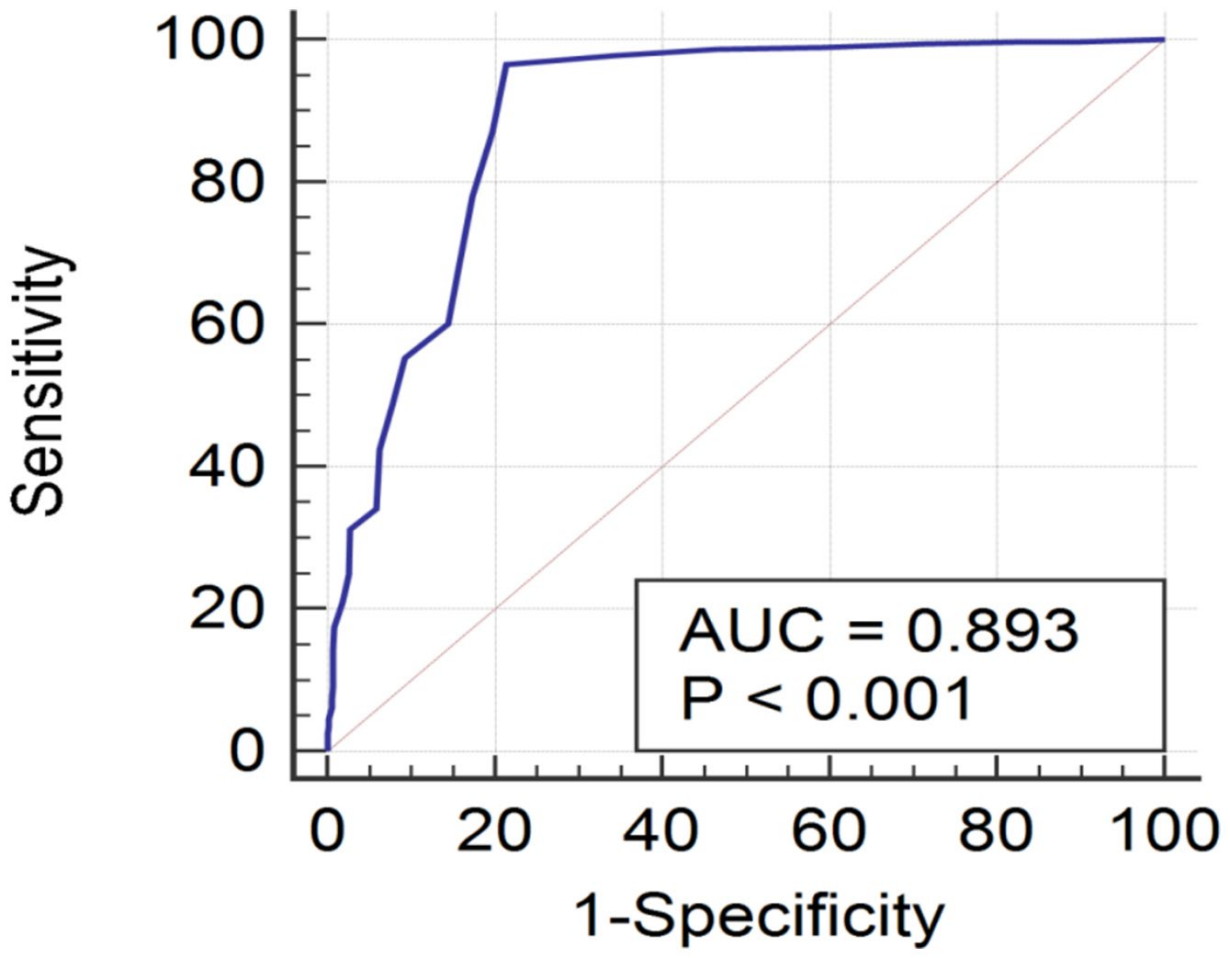
ROC curve of the MPAI using the high-risk class derived from latent class model as gold standard.

Using the binary outcomes (“non-case” and “case”) procured from LCA as the reference standard, the receiver operating characteristic (ROC) plot of the sensitivity versus 1-specificity of various MPAI scores revealed the area under the curve (AUC) of 0.893 (95% CI, 0.879 to 0.905, *p* < 0.001) (Figure 2). The diagnostic value indices for potential generated cut-off points from 8 to 40. The optimal cut-off value of MPAI was >14, which corresponded to the maximum Youden index (Youden index = 0.751). In this case, the sensitivity was 96.5%, specificity was 78.64%, PPV and NPV were 86.7 and 93.9%, respectively (Table 5). The positive group (defined as MPAI score > 14) included 59.13% of the participants.

**Table 5:** ROC analysis of the MPAI using the high-risk class derived from latent class model as gold standard.

Cut-off value	Sensitivity (100%)	Specificity (100%)	PPV (100%)	NPV (100%)	Youden index
≥8	100.000	0.000	59.100		
8	99.700	10.900	61.800	96.200	0.106
9	99.700	18.020	63.800	97.700	0.177
10	99.480	28.690	66.900	97.400	0.282
11	98.960	40.990	70.800	96.400	0.400
12	98.660	53.510	75.400	96.500	0.522
13	97.760	65.700	80.500	95.300	0.635
**14**	**96.500**	**78.640**	**86.700**	**93.900**	**0.751**
15	87.020	80.260	86.400	81.000	0.673
16	78.000	82.630	86.700	72.200	0.606
17	68.830	84.140	86.300	65.100	0.530
18	60.100	85.540	85.700	59.700	0.456
19	55.330	90.720	89.600	58.400	0.461
20	48.620	92.230	90.100	55.400	0.409
21	42.430	93.740	90.700	53.000	0.362
22	34.150	94.170	89.500	49.700	0.283
23	31.250	97.300	94.400	49.500	0.286
24	24.910	97.410	93.300	47.300	0.223
25	21.030	98.170	94.300	46.200	0.192
26	17.520	99.140	96.700	45.400	0.167
27	14.390	99.240	96.500	44.500	0.136
28	11.190	99.350	96.200	43.600	0.105
29	9.250	99.350	95.400	43.100	0.086
30	7.460	99.460	95.200	42.600	0.069
31	6.190	99.460	94.300	42.300	0.056
32	4.550	99.780	96.800	42.000	0.043
33	3.580	99.780	96.000	41.700	0.034
34	2.610	99.890	97.200	41.500	0.025
35	2.090	99.890	96.600	41.400	0.020
36	1.420	100.000	100.000	41.200	0.014
40	0.000	100.000		40.900	0.000

Bold value means best cut-off value.

### 5.5 Predicting mobile phone addiction LCA membership

A multinomial logistic regression is conducted with goal focus, interpersonal support emotional control, positive cognition, and family support as possible psychological resilience predictors of LCA membership as well as age, grade, academic performance, and family financial status as possible background characteristic predictors of LCA membership. As shown in Table 6, the multinomial logistic regression was employed by us to analyze Cluster normal mobile phone use, high-risk mobile phone use and mobile phone addiction as outcomes. Academic performance and grade negatively predicted memberships in cluster high-risk mobile phone use vs. normal mobile phone use and mobile phone addiction vs. normal mobile phone use. Goal focus, interpersonal support emotional control, positive cognition, and family support positively predicted memberships in cluster high-risk mobile phone use vs. normal mobile phone use, mobile phone addiction vs. normal mobile phone use, and mobile phone addiction vs. high-risk mobile phone use. Family financial status negatively predicted membership in cluster high-risk mobile phone use vs. normal mobile phone use, in contrast to cluster mobile phone addiction vs. high-risk mobile phone use.

**Table 6:** Multinomial logistic regression predicting LCA membership.

		*B*	*SE*	*P*	OR	95%CI	
						Low	Up
High-risk mobile phone use vs. Normal Mobile phone use
Age		−0.112	0.048	0.020	0.894	0.814	0.983
RSCA	Emotional control	−0.105	0.052	0.042	1.11	1.004	1.228
	Positive cognitive	−0.185	0.056	0.001	1.203	1.078	1.343
	RSCA Total	−0.089	0.047	0.060	0.915	0.834	1.004
Grade	Primary schools (6th)	−6.093	1.086	0.000	0.002	0.000	0.019
	Middle school (7th)	−6.422	0.805	0.000	0.002	0.000	0.008
	Middle school (8th)	−6.09	0.783	0.000	0.002	0.000	0.011
	Middle school (9th)	12.219	4000.037	0.998	202540.474	0.000	.c
	High school (1st)	−6.006	0.866	0.000	0.002	0.000	0.013
	High school (2st)	−6.582	0.769	0.000	0.001	0.000	0.006
	High school (3st)	0b					
Academic performance	Top 10	−1.017	0.364	0.005	0.362	0.177	0.739
	Above-average	−0.807	0.334	0.016	0.446	0.232	0.859
	Average	−1.116	0.334	0.001	0.327	0.170	0.63
	Below average	−0.578	0.328	0.079	0.561	0.295	1.068
	Bottom 10	0b					
Family financial status (LAINCOME, RMB)	≥8,000	−0.791	0.661	0.232	0.453	0.124	1.657
	6,000–7,999	−1.225	0.649	0.059	0.294	0.082	1.049
	4,000–5,999	−0.800	0.632	0.206	0.449	0.130	1.551
	2000–3,999	−0.799	0.621	0.198	0.450	0.133	1.52
	1,000–1999	−1.379	0.636	0.030	0.252	0.072	0.877
	600–999	−0.365	0.697	0.601	0.694	0.177	2.723
	200–599	0.363	0.766	0.636	1.437	0.320	6.446
	<200	0b					
Mobile phone addiction vs. normal mobile phone use
RSCA	Goal focus	−0.181	0.058	0.002	1.198	1.069	1.344
	Interpersonal support	−0.191	0.065	0.003	1.21	1.065	1.375
	Emotional control	−0.173	0.066	0.009	1.189	1.045	1.353
	Positive cognitive	−0.299	0.072	0.000	1.349	1.171	1.554
	Family support	−0.233	0.077	0.003	1.263	1.085	1.469
	RSCA Total	−0.206	0.061	0.001	0.814	0.722	0.918
Grade	Middle school (7th)	−7.654	0.886	0.000	0.000	0.835	0.003
	Middle school (8th)	−9.228	0.893	0.000	0.983	1.717	0.001
	Middle school (9th)	−5.227	5530.468	0.999	0.005	0.000	.c
	High school (1st)	−5.344	0.864	0.000	0.005	0.001	0.026
	High school (2st)	−3.178	0.752	0.000	0.042	0.010	0.182
	High school (3st)	0b					
Academic performance	Top 10	−0.61	0.422	0.148	0.543	0.238	1.242
	Above-average	−0.665	0.393	0.091	0.514	0.238	1.111
	Average	−1.237	0.385	0.001	0.290	0.137	0.617
	Below average	−0.722	0.391	0.065	0.486	0.226	1.046
	Bottom 10	0b					
Mobile phone addiction vs. high-risk mobile phone use
RSCA	Goal focus	−0.110	0.053	0.038	1.116	1.006	1.238
	Interpersonal support	−0.120	0.059	0.043	1.127	1.004	1.266
	Family support	−0.162	0.070	0.021	1.176	1.025	1.350
	RSCA total	−0.117	0.056	0.036	0.890	0.797	0.992
Gender	Male	0.440	0.170	0.010	1.553	1.112	2.169
	Female	0b					
Grade	Middle school (7th)	−1.232	0.451	0.006	0.292	0.121	0.705
	Middle school (8th)	−3.138	0.464	0.000	0.043	0.017	0.108
	Middle school (9th)	−16.446	2316.422	0.994	7.20E-08	0.000	.c
	High school (1st)	0.662	0.513	0.197	1.939	0.710	5.296
	High school (2st)	3.405	0.228	0.000	30.100	19.258	47.044
	High school (3st)	0b					
Family financial status (LAINCOME, RMB)	≥8,000	1.101	0.715	0.124	3.007	0.741	12.21
	6,000–7,999	1.403	0.678	0.039	4.066	1.076	15.369
	4,000–5,999	1.335	0.644	0.038	3.798	1.075	13.425
	2000–3,999	1.217	0.632	0.054	3.376	0.978	11.651
	1,000–1999	1.321	0.639	0.039	3.747	1.072	13.100
	600–999	1.127	0.687	0.101	3.087	0.803	11.862
	200–599	0.693	0.743	0.351	2.000	0.466	8.588
	<200	0b					

## 6 Discussion

Despite the growth in the incidence of mobile phone addiction among adolescents, many researchers tend to focus on potential risk factors contributing to mobile phone addiction, but few empirical studies have examined typologies of mobile phone addiction in a sample of Chinese adolescents. The current study empirically showed that a three-class model most effectively classified Chinese adolescents giving different degrees of problematic mobile phone usage. The three classes affected by the LCA were as follows: normal mobile phone use, high-risk mobile phone use, and mobile phone addiction. 21.25% of the mobile phone users fell into the category of normal, 32.1% of the mobile phone users were categorized as high-risk, and the rest (46.65%) were placed into the addiction group. When compared with the normal group, participants in the other two groups manifested more severe mobile phone-related problems. They also revealed a poorer psychosocial profile in the matter of stress, anxiety, depression, goal focus, interpersonal support, emotional control, and positive cognition.

The present study proposes that different classes of mobile phone users may be recognized through LCA, supplying a comprehensive view of the patterns of adolescents’ mobile phone use. This result was consistent with the findings of previous studies (39, 40). Given the fact that a mobile phone can provide more media resources than just a few years ago, mobile phone addiction may have more underlying groups. For this reason, personal characteristics may be a possible interpretation. Participants categorized in the LCA of mobile phone addiction were adolescents. The physical and psychological growth of adolescents is easily affected by many factors. The current study showed that academic performance, the education level of the mother, the educational level of the primary caregiver, the type of family education, family history of psychosis, stress, anxiety, depression, and psychological resilience were all related to mobile phone addiction of the adolescent participants. This result is consistent with findings of previous studies (10, 41, 42).

When compared with the high-risk mobile phone use group and the normal mobile phone use group, the result demonstrated that age, grade, psychological resilience and academic performance were negatively correlated with problematic mobile phone use. In other words, introducing mobile phones to children at a young age can lead to problematic mobile phone usage; the better the academic performance, the less likely an individual will develop problematic mobile phone usage. In this study, although grade was related to high-risk mobile phone use, middle school (9th grade) was not associated with high-risk mobile phone use. Due to the transition to higher education, 9th grade students have to deal with academic pressure, leading them to invest more time and energy into their studies.

Fischer-Grote et al. (43) suggested that gender, age, family, and personality might be risk factors for problematic smartphone use. Comparison between the mobile phone addiction group and the normal mobile phone use group revealed that psychological resilience and school grade were significantly negatively related to mobile phone addiction. There was a negative correlation between moderate academic performance and smartphone addiction. This study was consistent with previous research findings (7, 10, 44). Abd Rashid et al. (45) considered that an individual’s attention on academic study may be diverted if they have a severe addiction to their mobile phone. Bai et al. (46) regarded the effect of mobile phone addiction on academic rank was significant, pointing to a strong association between these two variables. When mobile phone addiction vs. high-risk mobile phone use, psychological resilience, gender, school grade (except for the 9th grade and the freshman year of high school), and family financial status had an effect on mobile phone addiction.

Liu et al. (47) considered that gender moderated the association between peer victimization and mobile phone addiction, with this association being stronger in girls than in boys. Korean girls were likely to more use their mobile phones and were at higher risk of mobile phone addiction (48). Borislav and Dijana (49) thought that age had a negative correlation with mobile phone addiction, while family risk factors were positively associated with disorder of early psychological development and mobile phone addiction. The subjective economic level, academic stress, and parental support were associated with a tendency to become addicted to the smartphone (50). In another study, Lee et al. (51) revealed that mobile phone addiction was related to higher levels of depression among low-income boys.

Based on the selected cut-off value, the prevalence of MPA among our sampled adolescents of Henan province in China was 59.13%. Alavi et al. (52) used the Mobile phone abuse questionnaire (MPA) to measure mobile phone usage of students, which showed that the cut-off point was 46 participants (31.2%) who were identified as having mobile phone addiction. The Smartphone Addition Inventory in Spanish (SPAI-Spain) was employed to investigate mobile addiction in a number of university communities in Spain. The result revealed that 23.67% of the participants were classified as dependent on the smartphone. As for gender, the percentage of women who suffered smartphone addiction accounted for 53.88% of the total (53). The literature review also revealed that the prevalence of smartphone addiction was 32% in Iran (54), 30.9% of middle school students in South Korea were categorized as a high-risk group (55), and 33.1% of secondary school students (56). Elsewhere, the rate of smartphone addiction among children and adolescents was 40.4% in 31 provinces of China (57), 46.1% of adolescents in Nigeria had moderate to high problematic phone usage (58), 71.9% of Saudi Arabian dental students had a mobile phone addiction (59), as did 85.4% of medical students in India (60). These variances could be partially assigned to the differences in participants and measurement tools. In this study, excessive use of mobile phone was due to the utilization of the virtual environment that was prevalent during the COVID-19 pandemic. At this time, many schools adopted online formats for teaching and various other educational activities, leading adolescents to primarily use smartphones or computers for remote learning. As a result, they spent more time on screens. Moreover, in China, socializing, shopping, recreation, and payment is now often done using social media such as TikTok, Wechat, QQ, interactive computer games, and so on, and this has become a principal factor of mobile phone addiction.

Based on these results, we suggest these cutoff point scores should be used as a threshold for normal mobile phone users as distinct from addicted mobile phone users. In research or diagnosis of mobile phone addiction, these results should be illustrated with care to sustain the validity of the questionnaire. Some consideration should be given when reporting cutoff value in interpretation, utilization of the cutoff value will rely on the purpose of the questionnaire and the popularity of mobile phone usage in the population under study. Moreover, no cutoff value is 100% accurate, so diagnostic errors will exist (61).

In sum, the current study used a sophisticated latent class analysis, and it highlights the differential associations between heterogeneous classes of multidimensional mental health and psychological resilience indicators for addictive mobile phone use in adolescents. Our discovery of the individual differences in factors that cause mobile phone addiction emphasizes the need to focus more on mobile phone users’ discrete psychological resilience and family status in future research. Further, the consideration of multidimensional pointers in our research spreads understanding of the united operation of various individual difference components in connection with varying degrees of risk for mobile phone addiction. Moreover, our results carry important real-world implications, in that suitable prevention or intervention methods for mobile phone addiction should target the particular psychological dimensions that are associated with mobile phone addiction, especially those that indicate a personal uneven psychological profile. For example, those whose latent classes are characterized by poor psychological resilience and high psychological distress will require stronger social support to improve their ability to regulate themselves psychologically. This appears to be a valid intervention device to reduce psychological dilemma with regards to addictive mobile phone use.

### 6.1 Limitations

This report must address several limitations of the study. The research used cross-sectional data. Given the nature of cross-sectional data, this study was unable to infer temporal sequence relationships, limiting its capacity for causal inferences and instead focused on correlation. The data were self-reported and may have been influenced by social desirability bias, despite efforts in this study to reduce bias through measures such as anonymity and absence of teachers. As this study drew on a relatively small number of schools in two cities in Henan province, the generalizations should be approached with caution. Therefore, in the future, more complicated longitudinal studies will be needed to establish the directionality of the relationship between these latent classes and mobile phone addiction. Future research should include more representative and diverse samples, as well as investigate the relationship between psychological characteristics and mobile phone addiction in other populations.

## 7 Conclusion

The current study discovered different patterns of problematic mobile phone use among adolescents living in Henan province. Three latent classes were recognized among the whole population. This is particularly important, as misuse of mobile phones is prevalent among adolescents, and ever-developing mobile phone technology may be perceived as a risk for increasing prevalence rates. Individual academic performance, parental education level, low psychological resilience, and a family history of mental illness appear to contribute to smartphone addiction amongst adolescents. In our study, we found that in the high-risk mobile phone addiction vs. normal mobile phone use, age, psychological resilience, school grade, and academic performance play a role in problematic phone usage. In the mobile phone addiction vs. normal smartphone use, psychological resilience, school grade, and academic performance influence mobile phone addiction. In the high-risk smartphone addiction vs. mobile phone addiction, psychological resilience, gender, and school grade contribute to smartphone addiction. The family’s economic status seems to have minimal impact on an adolescent’s mobile phone addiction. These results give a reliable basis to evaluate a lot of problems emanating from excessive mobile phone use. The increase in the proportion of mobile phone addiction among adolescents provides a rationale for implementing preventive measures and conducting further research.

## Data availability statement

The raw data supporting the conclusions of this article will be made available by the authors, without undue reservation.

## Ethics statement

The studies involving humans were approved by Ethics Committee of Nanyang Medical College (number: NYYZ20240001). The studies were conducted in accordance with the local legislation and institutional requirements. Written informed consent for participation in this study was provided by the participants’ legal guardians/next of kin.

## Author contributions

JW: Conceptualization, Writing – original draft. LJ: Investigation, Writing – review & editing. YL: Data curation, Investigation, Writing – review & editing. QL: Investigation, Writing – review & editing. YZ: Data curation, Investigation, Writing – review & editing. JZ: Data curation, Writing – review & editing. YJ: Data curation, Writing – review & editing. ZF: Conceptualization, Investigation, Supervision, Writing – review & editing.
